# Reports of Scottish Asylums

**Published:** 1918-04-06

**Authors:** T. C. Mackenzie

**Affiliations:** Medical Superintendent. District Asylum, Inverness


*To the Editor of* The Hospital.

### Reports of Scottish Asylums.

*To the Editor of* The Hospital.

Sir,—I have read with pleasure your leading article entitled "Reports of Scottish Asylums" in your issue of March 23, and should like to express to you my appreciation of the frank and generous amends it makes on the point to which my original letter referred, and also of its kindly references to myself. It is a further pleasure to note that you mention the name of Sir Thomas Clouston in relation to this subject. He made it a point in his annual reports to endeavour to awaken the public mind to the serious problems of mental disease, and he impressed their duty in this respect upon his assistant medical officers. I believe that much of the merit, which you acknowledge belongs to the Scottish Asylums, may be traced to his precept and example in this matter,—I am, yours truly,

T. C. Mackenzie,
Medical Superintendent.

District Asylum, Inverness, March 25, 1918.

